# Antioxidant Activity of Flavonoids in LPS-Treated IPEC-J2 Porcine Intestinal Epithelial Cells and Their Antibacterial Effect against Bacteria of Swine Origin

**DOI:** 10.3390/antiox9121267

**Published:** 2020-12-13

**Authors:** Dóra Kovács, Zita Karancsi, Orsolya Farkas, Ákos Jerzsele

**Affiliations:** Department of Pharmacology and Toxicology, University of Veterinary Medicine Budapest, 1078 Budapest, Hungary; kovacs.dora@univet.hu (D.K.); karancsi.zita@univet.hu (Z.K.); farkas.orsolya@univet.hu (O.F.)

**Keywords:** proanthocyanidins, grape seed extract, luteolin, flavonoids, polyphenols, oxidative stress, ROS, MIC, *Escherichia coli*, *Salmonella enterica* ser. Typhimurium

## Abstract

Beneficial effects of flavonoids are widely known in human medicine, but less information is available about their veterinary usage. Based on their antioxidant and antibacterial activity, proanthocyanidins (PAs) and luteolin (LUT) might be used in the prevention and treatment of gastrointestinal infections in swine. In this study, in vitro beneficial effects of grape seed oligomeric proanthocyanidins (GSOPs) and LUT were investigated against bacterial endotoxin (LPS)-induced oxidative stress in IPEC-J2 porcine epithelial intestinal cells. Furthermore, antibacterial effects of GSOP and LUT were assessed against field isolates of *Escherichia coli* and *Salmonella enterica* ser. Typhimurium. Both GSOP and LUT were found to possess potent in vitro antioxidant activity in LPS-treated IPEC-J2 cells; furthermore, they showed a bacteriostatic effect against the tested bacterial strains of porcine origin. Both flavonoids seem to be effective in the protection of porcine intestinal epithelial cells against Gram-negative bacteria in vitro, but further in vivo studies are necessary to confirm these activities and to establish their optimal dosage regimen for future usage in veterinary practice.

## 1. Introduction

Gastrointestinal (GI) infections are among the most significant health problems in pigs worldwide and are frequently caused by *Escherichia coli* and *Salmonella enterica* ser. Typhimurium [1]. *E. coli*, especially the enterotoxigenic pathotype (ETEC), can cause various diseases in swine, resulting in huge economic losses due to prevention and treatment related costs, decreased growth rate, or even death of animals. Infections by ETEC occur when predisposing environmental factors make pigs more susceptible to diseases—most common in neonatal and post-weaning ages [2]. Even though *Salmonella* strains often cause only asymptomatic infections in pigs, they pose a risk for human foodborne salmonellosis through the contamination of meat [3].

In the case of Gram-negative enteral infections, such as *E. coli* and *S*. Typhimurium, bacterial endotoxins (i.e., lipopolysaccharides, LPSs) are responsible for a significant part of the damage inflicted on the intestinal epithelium. LPS constitutes the outer cell membrane of Gram-negative bacteria [4] and is released when bacteria are killed by either the immune system or the applied bactericidal antibiotics. Harmful effects of endotoxins on the intestinal epithelium include morphological injury (e.g., mucosal necrosis), increased inflammation (through stimulation of proinflammatory cytokine release), mitochondrial damage (and consequential adenosine triphosphate (ATP) deficit) and alteration in the oxidative metabolism leading to oxidative stress [4,5,6,7,8]. Destruction inflicted by endotoxins impair the intestinal barrier integrity, leading to increased permeability, bacterial translocation and the absorption of toxins that can worsen prognosis of the disease [4,5,6,7].

Antibiotics are widely used in pigs for both the prevention and treatment of bacterial infections; however, resistant strains frequently occur due to inappropriate usage of these agents [2]. Alternative solutions, such as improved environmental hygienic measures, natural feed additives (e.g., antioxidants, immunostimulants), vaccines and genetic breeding of resistant herds are needed to address this problem worldwide [2,9].

Proanthocyanidins (PAs) are abundant plant polyphenols, present in a variety of plant foods, including fruits, legumes, nuts and cereals grains [10]. PAs are among the main biologically active components in cranberry, pomegranate, grape seed and lotus seed extracts. Structurally, they are oligomeric or polymeric flavan-3-ols, containing flavan-3-ol units linked together with C4-C8 or C4-C6 bonds (B-type), and sometimes with an additional C2-O7 linkage (A-type). They can be found in different molecular sizes, which are described by the degree of polymerization. Most common flavan-3-ol units include (epi)catechin, (epi)afzelechin and (epi)gallocatechin. PAs containing only (epi)catechin units are called procyanidins [11]. Different types of PAs can be found in various foods and can have diverse biological properties. For example, PAs extracted from grape seed only contain B-type linkages [12].

Proven effects of PAs include antioxidant, anti-inflammatory, antibacterial and antiviral activities [12]. Furthermore, PAs have shown antifungal properties by reducing adherence, biofilm formation and preformed biofilms of *Candida albicans* [13,14]. Their beneficial effects on intestinal inflammation and barrier integrity have also been reported in several cell culture and animal models of intestinal dysfunction [10]. Recently, the demand for PAs is increasing and there are numerous studies to prove their further beneficial effects on human health. PAs were shown to reduce the risk of hypertension and dyslipidemia [12], to cause apoptosis in several types of cancer cells [15] and to be potential candidates for ameliorating pathological processes in the GI tract caused by diet-induced obesity and inflammatory bowel disease [10].

Luteolin (LUT) is another biologically active polyphenol, belonging to the flavone group of flavonoids [16,17] that can be found in plants such as lettuce, celery, green pepper [16], cabbage, cauliflower and carrot [18]. The chemical structure of LUT consists of two phenolic and a pyranyl ring [17]. The molecule possesses several hydroxyl groups and a 2,3-double bond that have been associated with its biological activities [16]. LUT is known to have potent antioxidant and anti-inflammatory effects [18]; therefore, in traditional (human) Asian medicine, it was used for treating disorders associated with oxidative damage and acute inflammation [19]. It has also been demonstrated that LUT possess an anticarcinogenic effect by inhibiting tumor cell proliferation and inducing cell cycle arrest and apoptosis [16].

Based on their various beneficial activities, PAs and LUT might also be used as feed additives in veterinary medicine for the prevention and treatment of gastrointestinal infections. Their usage as antibiotic alternatives could contribute to the decrease in antibiotic consumption and consequently the development of resistance in bacteria. This is of highest importance in food-producing animals, such as pigs, as bacterial resistance developed in these species can spread to humans via several routes, including food of animal origin, contamination of the environment or direct animal–human contact [20]. It has been established that there is significant positive association between antibiotic usage in food-producing animals and bacterial resistance in humans [21]. *E. coli* and *Salmonella* are among those zoonotic bacteria that can be transmitted to humans by foodborne route [20] and therefore are able to confer resistance genes to bacteria in humans. Infections caused by resistant bacteria can lead to treatment failure [20] and are expected to become the major cause of death in humans by 2050 [22], which highlights public health importance of decreasing antibiotic usage in farm animals.

The aim of this study was to assess protective effects of grape seed oligomeric proanthocyanidins (GSOP) and LUT against LPS-provoked oxidative stress in IPEC-J2 porcine intestinal epithelial cells and their direct antibacterial effect on *E. coli* and *S*. Typhimurium field isolates of swine origin.

## 2. Materials and Methods

### 2.1. Chemicals and Equipment

All chemicals used in the cell culture experiments were purchased from Sigma-Aldrich (Darmstadt, Germany), including purified GSOP, LUT, dimethyl sulfoxide (DMSO), lipopolysaccharides (suitable for cell culture, derived from *Salmonella enterica* ser. Typhimurium, *Escherichia coli* O111:B4 and *E. coli* O127:B8), growth medium of IPEC-J2 cells, Neutral Red dye and 2′,7′-dichloro-dihydro-fluorescein diacetate (DCFH-DA). Mueller-Hinton liquid broth was ordered from Biolab Ltd. (Budapest, Hungary). All cell culture plates were obtained from Corning Inc. (Corning, New York, NY, USA), while microplates used for minimum inhibitory concentration (MIC) determination were supplied by VWR International (Radnor, PA, USA). The Biochrom EZ Read 400 Microplate Reader is manufactured by Biochrom Ltd. (Cambridge, UK), while Victor X2 2030 fluorometer is the product of PerkinElmer Inc. (Waltham, MA, USA). R software is developed by R Foundation (Vienna, Austria).

### 2.2. IPEC-J2 Cell Culture

IPEC-J2 is a nontransformed cell line, derived from the jejunal epithelium of a neonatal unsuckled piglet [23]. IPEC-J2 cell line was provided by Dr. Jody Gookin (Department of Clinical Sciences, College of Veterinary Medicine, North Carolina State University, Raleigh, NC, USA). Passage number of the used cells was around 50 for all experiments. Cells were cultured in the mixture of Dulbecco’s Modified Eagle’s Medium and Ham’s F-12 Nutrient (1:1, DMEM/F12), supplemented with fetal bovine serum (5%), insulin (5 μg/mL), transferrin (5 μg/mL), selenium (5 ng/mL), epidermal growth factor (5 ng/mL) and penicillin-streptomycin (1%). Culture conditions were set to 37 °C with 5% CO_2_. Cells were seeded onto 96- and 6-well polystyrene cell culture plates for the cell viability and the DCFH-DA assay, respectively. Experiments were started when the cells reached a differentiated, confluent monolayer.

### 2.3. Cell Viability Assay

Possible cytotoxic effect of purified GSOP and LUT at different concentrations and incubation time periods was tested with Neutral Red method based on the description of Repetto et al. [24]. GSOP was applied on IPEC-J2 cells at the concentrations of 50, 100 and 200 μg/mL, for 1, 12 and 24 h each. Similar incubation times were used in case of LUT, which was tested at 25, 50 and 100 μg/mL concentrations. Both GSOP and LUT were dissolved in plain DMEM/F12 medium for the experiment and applied on cells being cultured on 96-well plates. For the complete dissolution of LUT, DMSO was added to the working solutions at the maximum of 10% ratio. Effect of DMSO on cell viability alone has also been tested to exclude its potential influence on the results. Treatment with plain medium for 1 h was used as control in the experiment. Ratio of living cells was determined after the end of last treatments (24 h) by absorbance measurement with Biochrom EZ Read 400 Microplate Reader (on 540 nm wavelength).

### 2.4. Determination of IC ROS Levels

To provoke oxidative stress in the cell culture, all lipopolysaccharides (*S*. Typhimurium and both *E. coli*) were applied on IPEC-J2 cells at 10 µg/mL concentration [25]. For determination of their potential antioxidant activity, both GSOP and LUT were added to the cells in combination with all types of LPS each, the former at 50, 100 and 200 μg/mL, while the latter at 25, 50 and 100 μg/mL concentrations. Effects of both flavonoids on the amount of intracellular reactive oxygen species (IC ROS) alone were also tested at the same concentrations. All chemicals were dissolved in plain medium and were incubated with the cells for 1 h on 6-well plates. Similar to the cell viability assay, working solutions of LUT contained 10% DMSO at most therefore the effect of DMSO alone and in combination with LPS was also investigated. Cells treated only with plain medium served as control. To detect the amount of IC ROS, 10 µM DCFH-DA dye was used. IC ROS can oxidize DCFH-DA to a detectable fluorescent product, dichloro-fluorescein (DCF) [26]; therefore, elevated fluorescence values show increased amount of IC ROS. The method is not specific to certain types of ROS as various free radicals are able to oxidize DCFH-DA resulting in the quantification of overall oxidative stress in cells [26]. The dye was added to the cells for 60 min, followed by rinsing with medium, scraping and centrifugation for 10 min (at 3000× *g*). A Victor X2 2030 fluorometer was used to determine fluorescence of the samples (excitation wavelength: 480 nm, emission wavelength: 530 nm).

### 2.5. Bacterial Strains

The antibacterial effects (i.e., minimum inhibitory concentration, MIC) of GSOP and LUT were determined against 16 bacterial strains in total, including 8 *E. coli* and 8 *S*. Typhimurium strains isolated from the gastrointestinal tract or mesenteric lymph nodes of pigs. Strains were stored at −80 °C in Mueller-Hinton liquid broth (MH) supplemented with 20% sterile glycerol before the experiments. Twenty-four hours prior to MIC determination, bacteria were propagated in MH at 37 °C.

### 2.6. MIC Determination

For determination of the antibacterial activity of GSOP and LUT, the broth microdilution method was performed according to Clinical and Laboratory Standards Institute (CLSI) guideline M07-A10 [27]. GSOP and LUT were dissolved in DMSO for the investigation. Solutions, diluted two-fold, were prepared with MH broth on 96-well microtiter plates with the final concentrations of both flavonoids being set to 4096, 2048, 1024, 512, 256, 128, 64, 32, 16 and 8 μg/mL. Afterwards, 24 h cultures of bacterial strains were centrifuged for 10 min (at 3000× *g*), then washed and resuspended in physiological saline in order to achieve optical density of 0.1 at 600 nm, which is considered as equal to 10^8^ colony forming units (CFUs) in 1 mL of the suspension and a standard of 0.5 on the MacFarland scale. Bacterial suspensions were then diluted to 10^6^ CFU/mL and spread on agar plates for control CFU counting. Inoculation of the suspensions on plates containing GSOP or LUT resulted in a final 10^5^ CFU/mL concentration of bacteria. This was followed by 24 h of incubation at 37 °C and the evaluation of MIC values with the unaided eye. After determination of the MIC for each strain separately, MIC50 and MIC90 values (i.e., MIC that inhibits 50 or 90% of isolates, respectively) were calculated for both *E. coli* and *Salmonella*.

### 2.7. Statistics

Statistical analysis of data obtained in the cell culture experiments was performed with R 3.3.2 (2016) software (R Foundation for Statistical Computing, Vienna, Austria). Differences among the mean values of different experimental groups were evaluated with one-way ANOVA and Tukey post-hoc test. Results were interpreted as significant if the *p* value was lower than 0.05. No statistical analysis was performed in case of the MIC determination.

## 3. Results

### 3.1. Cell Viability Assay

**Figure 1:**
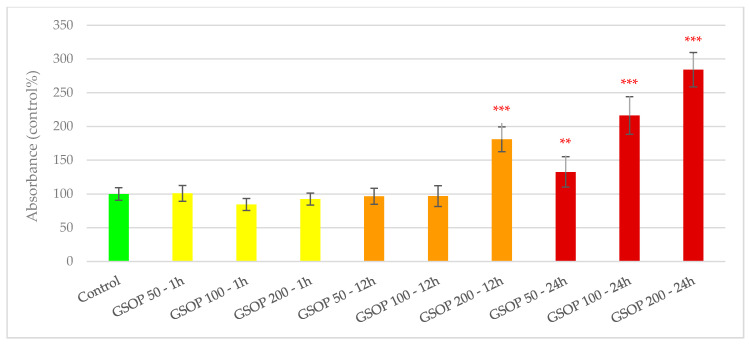
Viability of IPEC-J2 cells after treatment with purified grape seed oligomeric proanthocyanidins (GSOPs). Control—plain cell culture medium treatment for 1 h; GSOP 50—1 h: 50 µg/mL GSOP treatment for 1 h; GSOP 100—1 h: 100 µg/mL GSOP treatment for 1 h; GSOP 200—1 h: 200 µg/mL GSOP treatment for 1 h; GSOP 50—12 h: 50 µg/mL GSOP treatment for 12 h; GSOP 100—12 h: 100 µg/mL GSOP treatment for 12 h; GSOP 200—12 h: 200 µg/mL GSOP treatment for 12 h; GSOP 50—24 h: 50 µg/mL GSOP treatment for 24 h; GSOP 100—24 h: 100 µg/mL GSOP treatment for 24 h; GSOP 200—24 h: 200 µg/mL GSOP treatment for 24 h. Data are shown as means with standard deviations, *n* = 6/group. Significant difference compared to the untreated control: ** *p* < 0.01, *** *p* < 0.001; in red: higher values than control.

Purified GSOP did not show any toxic effect on viability of IPEC-J2 cells in any of the applied concentrations and treatment durations. Measured absorbance values, which show correlation with the amount of viable cells, did not differ significantly between the untreated control and GSOP-treated cells in case of the shortest treatment period (1 h, with all concentrations) and in case of 50 and 100 µg/mL GSOP applied for 12 h. GSOP 200 µg/mL treatment for 12 h and all concentrations given for 24 h resulted in significantly increased absorbance values compared to the control (*p* < 0.001 in all cases except GSOP 50 µg/mL for 24 h: *p* < 0.01). Results of GSOP in the cell viability assay are shown in Figure 1.

**Figure 2:**
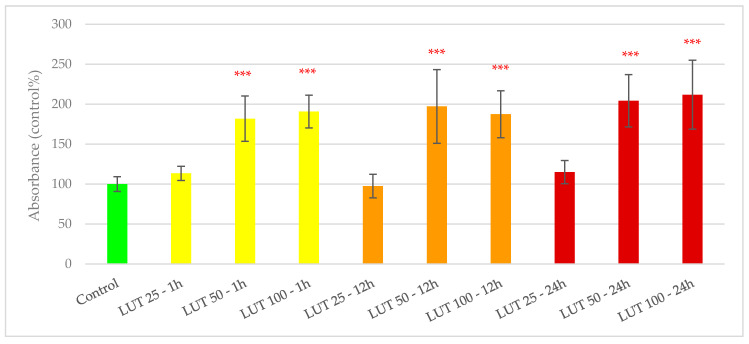
Viability of IPEC-J2 cells after treatment with luteolin (LUT). Control—plain cell culture medium treatment for 1 h; LUT 25—1 h: 25 µg/mL LUT treatment for 1 h; LUT 50—1 h: 50 µg/mL LUT treatment for 1 h; LUT 100—1 h: 100 µg/mL LUT treatment for 1 h; LUT 25—12 h: 25 µg/mL LUT treatment for 12 h; LUT 50—12 h: 50 µg/mL LUT treatment for 12 h; LUT 100—12 h: 100 µg/mL LUT treatment for 12 h; LUT 25—24 h: 25 µg/mL LUT treatment for 24 h; LUT 50—24 h: 50 µg/mL LUT treatment for 24 h; LUT 100—24 h: 100 µg/mL LUT treatment for 24 h. Data are shown as means with standard deviations, *n* = 6/group. Significant difference compared to the untreated control: *** *p* < 0.001; in red: higher values than control.

Similar to GSOP, treatment with LUT did not have any negative influence on viability of IPEC-J2 cells. Treatment with 25 µg/mL LUT did not significantly alter absorbance (and therefore ratio of viable cells in the culture) regardless of treatment duration. However, all other treatments (i.e., higher concentrations of LUT applied for 1, 12 and 24 h) could significantly increase measured absorbance values (*p* < 0.001). Results of LUT in the cell viability assay are shown in Figure 2.

### 3.2. IC ROS Levels

**Figure 3:**
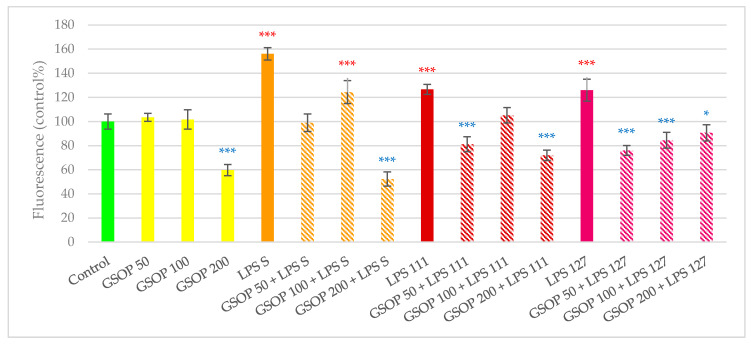
Amount of intracellular reactive oxygen species after treatment with bacterial endotoxin (LPS), purified grape seed oligomeric proanthocyanidins (GSOPs) and their combinations. Control—plain cell culture medium treatment; GSOP 50: 50 µg/mL GSOP; GSOP 100: 100 µg/mL GSOP; GSOP 200: 200 µg/mL GSOP; LPS S: *S*. Typhimurium endotoxin 10 µg/mL; GSOP 50 + LPS S: 50 µg/mL GSOP + *S*. Typhimurium endotoxin 10 µg/mL; GSOP 100 + LPS S: 100 µg/mL GSOP + *S*. Typhimurium endotoxin 10 µg/mL; GSOP 200 + LPS S: 200 µg/mL GSOP + *S*. Typhimurium endotoxin 10 µg/mL; LPS 111: *E. coli* O111:B4 endotoxin 10 µg/mL; GSOP 50 + LPS 111: 50 µg/mL GSOP + *E. coli* O111:B4 endotoxin 10 µg/mL; GSOP 100 + LPS 111: 100 µg/mL GSOP + *E. coli* O111:B4 endotoxin 10 µg/mL; GSOP 200 + LPS 111: 200 µg/mL GSOP + *E. coli* O111:B4 endotoxin 10 µg/mL; LPS 127: *E. coli* O127:B8 endotoxin 10 µg/mL; GSOP 50 + LPS 127: 50 µg/mL GSOP + *E. coli* O127:B8 endotoxin 10 µg/mL; GSOP 100 + LPS 127: 100 µg/mL GSOP + *E. coli* O127:B8 endotoxin 10 µg/mL; GSOP 200 + LPS 127: 200 µg/mL GSOP + *E. coli* O127:B8 endotoxin 10 µg/mL. Data are shown as means with standard deviations, *n* = 6/group. Significant difference compared to the untreated control: * *p* < 0.05, *** *p* < 0.001; in red: higher values, in blue: lower values than control.

Treatment with all three lipopolysaccharides caused oxidative stress in IPEC-J2 cells, resulting in significantly increased amount of intracellular ROS compared to the untreated control (*p* < 0.001). GSOP alone either did not change (50 and 100 µg/mL) or significantly decrease (200 µg/mL, *p* < 0.001) the amount of IC ROS in IPEC-J2 cells. When GSOP was combined with LPS, GSOP was able to alleviate harmful effect of the endotoxin in all combinations (i.e., all concentrations of GSOP combined with all types of LPS) except GSOP 100 µg/mL + *S*. Typhimurium LPS. In most cases, IC ROS levels of the combinations were similar to the control (GSOP 50 µg/mL + *S*. Typhimurium LPS; GSOP 100 µg/mL + *E. coli* O111:B4 LPS) or significantly lower (GSOP 200 µg/mL + *S*. Typhimurium LPS; GSOP 50 µg/mL + *E. coli* O111:B4 LPS; GSOP 200 µg/mL + *E. coli* O111:B4 LPS; GSOP in all concentrations + *E. coli* O127:B8 LPS). Results of GSOP in the DCFH-DA assay are shown in Figure 3.

**Figure 4:**
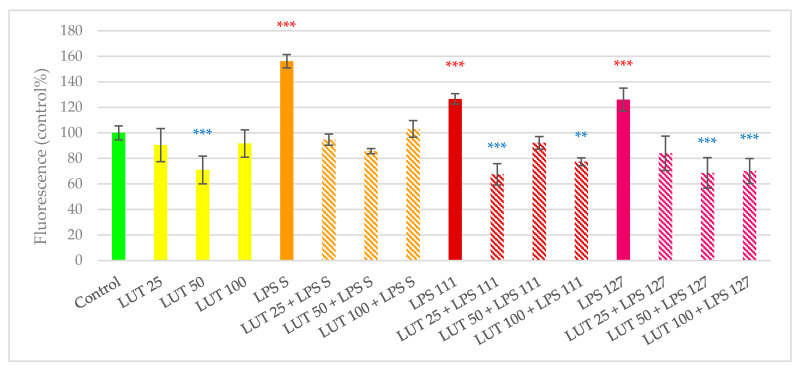
Amount of intracellular reactive oxygen species after treatment with bacterial endotoxin (LPS), luteolin (LUT) and their combinations. Control—plain cell culture medium treatment; LUT 25: 25 µg/mL LUT; LUT 50: 50 µg/mL LUT; LUT 100: 100 µg/mL LUT; LPS S: *S*. Typhimurium endotoxin 10 µg/mL; LUT 25 + LPS S: 25 µg/mL LUT + *S*. Typhimurium endotoxin 10 µg/mL; LUT 50 + LPS S: 50 µg/mL LUT + *S*. Typhimurium endotoxin 10 µg/mL; LUT 100 + LPS S: 100 µg/mL LUT + *S*. Typhimurium endotoxin 10 µg/mL; LPS 111: *E. coli* O111:B4 endotoxin 10 µg/mL; LUT 25 + LPS 111: 25 µg/mL LUT + *E. coli* O111:B4 endotoxin 10 µg/mL; LUT 50 + LPS 111: 50 µg/mL LUT + *E. coli* O111:B4 endotoxin 10 µg/mL; LUT 100 + LPS 111: 100 µg/mL LUT + *E. coli* O111:B4 endotoxin 10 µg/mL; LPS 127: *E. coli* O127:B8 endotoxin 10 µg/mL; LUT 25 + LPS 127: 25 µg/mL LUT + *E. coli* O127:B8 endotoxin 10 µg/mL; LUT 50 + LPS 127: 50 µg/mL LUT + *E. coli* O127:B8 endotoxin 10 µg/mL; LUT 100 + LPS 127: 100 µg/mL LUT + *E. coli* O127:B8 endotoxin 10 µg/mL. Data are shown as means with standard deviations, *n* = 6/group. Significant difference compared to the untreated control: ** *p* < 0.01, *** *p* < 0.001; in red: higher values, in blue: lower values than control.

LUT treatment alone on IPEC-J2 cells at 25 and 100 µg/mL did not influence IC ROS production; however, 50 µg/mL LUT treatment resulted in a significantly lower ROS level compared to the control (*p* < 0.001). A possible explanation of this finding is that there might be nonlinear dose effects of flavonoids as stated by Kay et al. [28]. All concentrations of LUT showed potent antioxidant activity against both *E. coli* and *S.* Typhimurium LPS treatments. IC ROS amount of the cells treated with LUT + LPS combinations were either similar (all concentrations of LUT + *S.* Typhimurium LPS; LUT 50 µg/mL + *E. coli* O111:B4 LPS; LUT 25 µg/mL + *E. coli* O127:B8 LPS) or significantly lower (*p* < 0.01 or lower) than the untreated control (LUT 25 µg/mL + *E. coli* O111:B4 LPS; LUT 100 µg/mL + *E. coli* O111:B4 LPS; LUT 50 µg/mL + *E. coli* O127:B8 LPS; LUT 100 µg/mL + *E. coli* O127:B8 LPS). The applied *Salmonella* LPS resulted in a higher IC ROS production increase compared to LPS of *E. coli* origin. As a consequence, antioxidant activity of LUT in combination with *Salmonella* LPS could reduce IC ROS amount to the control level, while when applied together with *E. coli* LPS, LUT was able to decrease IC ROS level below the control values. Results of LUT in the DCFH-DA assay are shown in Figure 4.

### 3.3. Antibacterial Activity

In this study, antibacterial activity of GSOP against the investigated *E. coli* and *S*. Typhimurium strains was found only at high concentrations. GSOP could inhibit growth of all isolates with a MIC of 2048 µg/mL. As the bacteriostatic activity of GSOP was observed at the same concentration in the case of all isolates, obtained MIC50 and MIC90 values of GSOP were both 2048 µg/mL. However, LUT showed a more potent bacteriostatic effect and was able to inhibit all tested bacteria at 256 µg/mL concentration (MIC). Similar to GSOP, MIC of LUT was similar against all strains; therefore, MIC50 and MIC90 values of LUT proved to be 256 µg/mL against the investigated *E. coli* and *Salmonella* field isolates. Obtained MIC50 and MIC90 values are shown in Table 1.

## 4. Discussion

Antimicrobial resistance is among the major health challenges of the 21st century, affecting human and animal health in an interdependent way [29]. Usage of antibiotics in food-producing animals, such as pigs, is related to higher prevalence of resistant bacteria (e.g., *E. coli* and *Salmonella*) in both animals and humans [30]. In order to reduce antibiotic consumption in farm animals, research for alternative strategies that promote gastrointestinal health is of utmost importance [3]. Potential feed additives that can be used for growth promotion and enhancing gut health include—without claim for completeness—pre-, pro- and synbiotics, organic acids, antimicrobial peptides, bacteriophages and phytochemicals (mainly plant polyphenols) [31].

To the best of our knowledge, this study was the first to investigate effects of GSOP and LUT on the IPEC-J2 porcine intestinal epithelial cell line. We demonstrated that treatment with GSOP up to 200 µg/mL and 24 h did not have any negative effect on viability of IPEC-J2 cells. This result is in accordance with the findings of Yan et al. [32], who reported absence of cytotoxicity when IPEC-J2 cells were treated with different procyanidins and their degradation products. Furthermore, Chedea et al. [33] also found that addition of aqueous grape pomace extract (containing several procyanidins) to IPEC-1 culture did not alter number of viable cells. LUT could also be used safely on IPEC-J2 cells up to 100 µg/mL concentration for 24 h. This is the first study to show that LUT has no harmful effects on the viability of porcine intestinal epithelial cells. In the cell viability assay, we could also observe elevated absorbance values after some of the treatments with GSOP and LUT, which suggests an increase in the number of viable cells. Similar to our results, a cell viability increase could be observed after PA [34] and LUT [35] treatments in some cell cultures. Galarraga-Vinueza et al. [34] suggested that this effect might be due to antioxidant and other bioactive properties of PAs. However, absence of significant alteration in cell viability after treatment with these flavonoids is also reported in several cell types [32,33,36,37]. Possible reasons behind these observations are the differences in the used cells, or concentration and source of the tested substances. However, further research is needed to reveal the exact underlying mechanisms. Inclusion of DMSO up to 10% in working solutions do not alter the ratio of viable cells in IPEC-J2 culture (data not shown).

The antioxidant activities of different PAs are widely known [12] and have previously been demonstrated both in vitro and in vivo against several oxidative stress inducer factors. In pigs, antioxidant effects of PAs have mostly been investigated in in vivo experimental settings. Chedea et al. [33] tested the effects of 5% grape pomace supplementation in the diet of piglets on the antioxidant status of their GI tract. Inclusion of grape pomace in feed of piglets resulted in an increase in antioxidant enzyme activity and the total antioxidant status, while a decrease in lipid peroxidation. Taranu et al. [38] provoked oxidative stress in piglets by contaminating their feed with aflatoxin B1 and assessed protective effect of 8% grape seed meal addition to the diet. Aflatoxin elevated the level of lipid peroxidation and caused a reduction in antioxidant enzyme capacity in the GI tract, liver and systemic circulation as well, the effects of which could be ameliorated by dietary inclusion of grape seed meal. Increased total antioxidant capacity and antioxidant enzyme activity were also observed by Hao et al. [39] when feed of pigs was supplemented with 100 and 150 mg/kg grape seed procyanidins. In contrast, Gessner et al. [40] did not observe alterations in antioxidant capacity of pigs that were fed with a commercial feed additive containing grape seed and grape marc meal in 1% of their diet. Our results supplement the above-mentioned information with in vitro findings on a porcine derived cell line. We were able to demonstrate in vitro that purified GSOP could decrease IC ROS production of IPEC-J2 porcine intestinal epithelial cells in LPS-provoked oxidative stress. GSOP was effective against LPS of both *E. coli* and *S.* Typhimurium origin. Protective effect of PAs against LPS-induced oxidative stress has been previously described in human Caco-2 cells [41] and in Wistar rats in vivo [42], but our study was the first to assess it in cells of porcine origin.

The protective effect of LUT against oxidative stress has been previously demonstrated in various cell types, including liver tissues exposed to lead acetate [43], LPS-stimulated macrophages [44] and cisplatin-damaged kidney cells [45]. Furthermore, the antioxidant activity of LUT has been suggested to correlate with its anticancer effect in human colon cancer cells [46] and human lung squamous carcinoma cells [47]. In terms of LPS-induced cellular damage, studies mainly focus on anti-inflammatory properties of LUT, which has been demonstrated in LPS-treated hepatoma cells [48], macrophages [44,49], microglia [50], intestinal epithelial cells and dendritic cells [51]. It is suggested that antioxidant activity of LUT plays a role in the observed anti-inflammatory effects [48]. In pigs, antioxidant properties of LUT have not been previously tested either in vitro or in vivo. In our study, we assessed the effect of LUT on ROS production from a different approach compared to previous investigations as we used the context of intestinal infections caused by Gram-negative bacteria. For the first time, we were able to prove the antioxidant effect of LUT in *E. coli* and *Salmonella* LPS-induced porcine intestinal epithelial cell lines. Our finding is in accordance with the above-mentioned reports on different cell cultures and tissues. The DMSO content of working solutions up to 10% do not interfere with IC ROS production of IPEC-J2 cells in LPS-induced oxidative stress (data not shown).

The antibacterial effect of PAs can be highly affected by their structure. PA content is suggested to have a relevant role in the efficacy of cranberry in prevention and treatment of urinary tract infections, which are mostly caused by uropathogenic *E. coli* (UPEC) strains [52]. This effect mostly relies on antiadhesive action that prevents bacterial colonization of the urinary tract and has been described about A-type PAs but was not observed in the case of B-type structures [52,53]. Regarding efficacy against gastrointestinal *E. coli* infections, Tang et al. [54] reported that B-type oligomeric procyanidins (extracted from lotus seedpod) could inhibit growth of two ETEC strains at 800 and 1200 µg/mL concentrations and showed bactericidal effect at 1500 µg/mL. Against the same bacteria, A-type oligomeric procyanidins (source: litchi pericarp) were only bacteriostatic at 2500 and 3500 µg/mL, while bactericidal at 3000 and 4500 µg/mL concentrations [54]. In the work of Levy et al. [55], grape seed extract (containing B-type procyanidins) had significantly lower MIC values against *Listeria monocytogenes* and *S*. Typhimurium, but showed similar inhibitory effect against *E. coli* than peanut skin extract (rich in A-type procyanidins). Alshaibani et al. [56] investigated the effect of cranberry PAs against seven enteropathogenic *E. coli* (EPEC) and six ETEC strains, and reported 18,000 µg/mL as the MIC, and a minimum bactericidal concentration (MBC) of 36,000 µg/mL of the extract. However, the low number of investigated bacterial strains limits interpretation of the above-mentioned study results. In the current study, we investigated the antibacterial effect of GSOP against 8-8 field isolates of *E. coli* and *S*. Typhimurium and could detect its bacteriostatic activity at a high, 2048 µg/mL concentration, which is in correlation with the above-mentioned data.

Reported MIC values of LUT against *E. coli* and *Salmonella* strains vary in the literature. Wouamba et al. [57] reported a low MIC of 3.12 µg/mL against *E. coli* and 6.25 µg/mL against *S*. Typhimurium; however, only one reference strain per each bacterial species was tested in their study. MIC values of 64 µg/mL were obtained by Qian et al. [58] when they tested the activity of LUT against four strains of *E. coli*, including one ATCC strain and three isolates from raw pork meat. Adamczak et al. [59] have also investigated antibacterial effect of LUT against four *E. coli* strains, but they could only find inhibitory activity at higher concentrations (MIC: 500 µg/mL). However, similarly to the results of PAs, a low number of investigated strains might be the reason behind these controversial results. In our study, we could observe bacteriostatic effect of LUT at 256 µg/mL concentration against 8-8 field isolates of *E. coli* and *S*. Typhimurium. This result is in the midline compared to previously reported MIC values and can broaden the knowledge about the antibacterial effect of LUT with a higher number of strains involved in the investigation.

## 5. Conclusions

In this study, for the first time, we demonstrated an absence of cytotoxicity of purified grape seed oligomeric proanthocyanidins and luteolin on an IPEC-J2 porcine intestinal epithelial cell line. We were able to prove in vitro antioxidant activity of GSOP and LUT against LPS-induced oxidative stress in the same cells. LUT could inhibit growth of *E. coli* and *S*. Typhimurium field isolates with a MIC of 256 µg/mL, while bacteriostatic effect of GSOP was only observed at higher (2048 µg/mL) concentrations. The tested flavonoids are promising potential feed additives for swine, but further in vivo studies are necessary to confirm their beneficial effects and optimal dosing regimen. Future usage of these substance in food-producing animals might contribute to the decrease in antibiotic consumption and consequently the development of resistance against antibiotics.

## Figures and Tables

**Table 1:** Minimum inhibitory concentration (MIC) values of grape seed oligomeric proanthocyanidins (GSOPs) and luteolin (LUT) against the tested bacterial strains. Values are expressed as μg/mL, *n* = 8 strains for both bacterial species.

Bacteria	GSOP		LUT	
	MIC50	MIC90	MIC50	MIC90
*E. coli*	2048	2048	256	256
*S.* Typhimurium	2048	2048	256	256

## Data Availability

All data that support the above-detailed findings can be obtained from the corresponding author upon request.
